# A study on the role of cuproptosis-related immune checkpoint genes in non-small cell lung cancer

**DOI:** 10.1097/MD.0000000000047163

**Published:** 2026-01-16

**Authors:** Ke Han, Ju Kun Wang, Jing Yao

**Affiliations:** a Department of Thoracic Surgery, Beijing Jishuitan Hospital, Capital Medical University, Beijing, China; b Department of General Surgery, Xuanwu Hospital of Capital Medical University, Beijing, China; c Cerebrovascular Disease Department, Neurological Disease Center, Beijing Anzhen Hospital, Capital Medical University, Beijing, China.

**Keywords:** cuproptosis-related immune checkpoint genes, non-small cell lung cancer, prognosis, risk model

## Abstract

Non-small cell lung cancer (NSCLC) is a common malignancy. Studies have demonstrated the crucial role of cuproptosis and immune checkpoint genes (ICGs) in the process of cancer progression, while it remains unclear whether cuproptosis-related immune checkpoint genes (CRICGs) can help predict the prognosis of NSCLC and provide guidance for establishing appropriate treatment. Thirteen cuproptosis-related genes and 79 ICGs were obtained for correlation analyses to determine the expression levels of CRICGs. Univariate Cox and least absolute shrinkage and selection operator regression analyses were conducted to identify prognosis-related CRICGs and develop a predictive model for the prognosis of NSCLC. Four CRICGs were selected to construct the predictive model, followed by an assessment of its performance. The risk score of the predictive model was deemed to be an independent prognostic factor in NSCLC. Gene set enrichment analyses showed that CRICGs were enriched in cancer-related pathways. The prognostic nomogram based on the risk scores and clinical data of NSCLC patients could accurately predict 3- and 5-year survival. The high- and low-risk groups showed significant differences in immune cell infiltration and ICG expression levels. The results of drug susceptibility testing of NSCLC to conventional targeted therapy and chemotherapy suggested a higher probability of drug resistance in the high-risk group. Finally, 8 CRICG-targeting small-molecule drugs were found to have therapeutic potential for NSCLC.

## 1. Introduction

Primary lung cancer is currently the most common malignancy and one of the leading health threats at the global level,^[1]^ and non-small cell lung cancer (NSCLC) makes up about 85% of all lung cancer cases. Although early diagnosis and treatment can provide favorable survival outcomes, only about 15% of NSCLC cases are identified at an early stage. Among patients with advanced NSCLC, especially those with metastases, the 5-year survival is <4%.^[2]^ Although the individual prognosis largely depends on the specific tumor stage, some with early-stage NSCLC can experience early recurrence or metastasis, while those with advanced NSCLC may live much longer than expected. Therefore, comprehensive and individualized analyses are required in addition to tumor-node-metastasis (TNM) staging as the major prognostic tool to accurately define a patient’s prognosis and develop tailored treatment options. Active efforts have been made to discover prognostic factors complementary to the TNM system, such as the identification of new prognostic biomarkers, and the construction of an immune cell infiltration (ICI) score model.^[3]^

Cuproptosis is a new kind of copper-induced cell death distinct from the more well-known forms of programmed and nonprogrammed cell death including apoptosis, pyroptosis, autophagy, and ferroptosis, and dependent on mitochondrial respiration. This copper-triggered cell death occurs when copper binds directly with lipoylated components of the tricarboxylic acid cycle, which results in lipoylated protein aggregation and subsequent iron–sulfur cluster protein loss that cause proteotoxic stress and ultimately cell death. Recent studies have shown that copper levels are significantly elevated in the serum and tumor tissue of cancer patients as compared with the healthy controls^[4,5]^; if not properly regulated to maintain homeostasis, copper can be cytotoxic and affect cancer development and progression.^[6]^ Copper-dithiocarbamate and -disulfiram complexes, and copper-chelating agents such as tetrathiomolybdate and trientine are applied to anticancer therapy based on the copper homeostasis mechanism.^[7,8]^

Immune checkpoint genes (ICGs) are responsible for balancing stimulatory and inhibitory pathways and are crucial for maintaining self-tolerance and modulating the type, intensity, and duration of immune responses.^[9]^ Mounting evidence shows that in cancer, immune checkpoint pathways are activated to suppress immunological activity against tumors and inhibit immune responses.^[10]^ Immune checkpoint blockade represents a type of epoch-making immunotherapy that uses various antibodies to block immune checkpoint pathways.

Although cuproptosis and immune checkpoints are high on the priority list of cancer research, little has been done to investigate whether or not cuproptosis-related immune checkpoint genes (CRICGs) can help predict the prognosis and guide the treatment of NSCLC. In this study, a predictive model was established with 4 CRICGs that were identified with bioinformatics approaches, and a prognostic nomogram was developed using the risk scores generated from the predictive model, which was found to effectively predict the prognosis of patients with NSCLC. Besides, the study results suggested correlations between the 4 CRICGs and ICI levels in the tumor microenvironment (TME). Eight small-molecule drugs (SMDs) were further selected to treat NSCLC by targeting these CRICGs.

## 2. Materials and methods

### 2.1. Data collection

The training dataset included mRNA expression and clinical data of tumor tissue samples from NSCLC patients (including lung adenocarcinoma [LUAD] and lung squamous cell carcinoma [LUSC]) available on The Cancer Genome Atlas (TCGA) data portal (https://portal.gdc.cancer.gov/). The validation dataset was comprised of GSE30219 downloaded from the Gene Expression Omnibus (GEO) database (https://www.ncbi.nlm.nih.gov/geo/). Cuproptosis-related genes (CRGs; n = 13) were obtained from a published work,^[11]^ and ICGs (n = 79) were from another study.^[12]^ Expression levels of these CRGs and ICGs were determined via R-4.2.0, followed by a correlation analysis using the “limma” package with the correlation coefficient ≥ 0.3 and *P* < .05 to quantify the expression levels of CRICGs.

### 2.2. Gene set enrichment analysis (GSEA) and protein–protein interaction (PPI)

The CRICGs underwent gene ontology (GO) and Kyoto Encyclopedia of Genes and Genomes (KEGG) enrichment analyses using the “clusterProfiler,” “org.Hs.eg.db,” “ggplot2,” and ‘enrichplot’ packages. A false discovery rate or *P*-value < .05 was defined as significant enrichment. The STRING database (http://www.string-db.org/) was utilized to predict protein–protein interaction (PPI) and draw PPI networks in combination with Cytoscape.

### 2.3. Development and performance evaluation of the predictive model based on cuproptosis-related immune checkpoint genes (CRICGs)

The “survival” R package was employed to analyze and screen for CRICGs of prognostic value. Subsequently, a univariate Cox regression analysis was performed on the prognosis-related CRICGs. Then, a least absolute shrinkage and selection operator (LASSO)-penalized Cox regression analysis was carried out using the “glmnet” package to develop a prognostic risk model. The risk score can be calculated via the following formula:


$$\begin{array}{l} \;\;\;\begin{array}{l} Risk\;\;\;score=\left(Coef_{1}\times expression\;\;\;of\;\;\;mRNA_{1}\right)+\left(Coef_{2}\times expression\;\;\;of\;\;\;mRNA_{2}\right)\;+\ldots \ldots +\left(Coef_{n}\times expression\;\;\;of\;\;\;mRNA_{n}\right)\; \end{array} \end{array}$$

Wherein Coef_1, 2, 3, …, n_ stand for coefficients of mRNA_1, 2, 3, …, n_ in the LASSO Cox regression model. The NSCLC patients were divided into high- and low-risk groups by the median risk score (median cutoff). In the survival analysis, a Kaplan–Meier (KM) curve was depicted to compare the prognosis between the high- and low-risk groups. The “survivalROC” package was utilized to compute a receiver operating characteristic (ROC) curve for performance evaluation of the prognostic risk model. The predictive performance was further evaluated using NSCLC samples from the GEO database as the validation dataset.

### 2.4. Construction of prognostic nomogram

Uni- and multivariate Cox regression analyses of the risk scores and clinical data of the NSCLC patients were performed using the “survival” package to measure the prognostic value of such risk scores. The “pheatmap” package, chi-square test, and Fisher’s test were implemented to verify whether the CRICGs used for constructing the risk model were involved in the development and progression of NSCLC. The “regplot” and “rms” packages were utilized to plot a prognostic nomogram of NSCLC based on the risk scores and clinical data for 3- and 5-year survival analysis. A calibration curve was created to assess the predictive performance of the prognostic nomogram.

### 2.5. Gene set enrichment analyses

Gene set enrichment analysis (GSEA) was conducted using the “enrichplot,” “org.Hs.eg.db,” “ggplot2,” and ‘clusterProfiler’ packages to explore potential molecular mechanisms in the high- and low-risk groups. Statistical significance was set at *P* < .05 or false discovery rate < 30%.

### 2.6. Analysis of immune cell infiltration (ICI) in the tumor microenvironment (TME)

Seven algorithms, including tumor immune estimation resource (TIMER), xCell, CIBERSORT, CIBERSORT-ABS, MCP-counter, quanTIseq, and EPIC, were integrated to analyze the differences between the high- and low-risk groups in ICI levels in the TME. Following that, the “ggpubr” package was applied to quantify the expression levels of 47 common ICGs – which were obtained from a previous study – in the high- and low-risk groups^[13]^ (given that CRICGs are already known to play a role in the immune response to NSCLC, is it necessary to measure the expression levels of these ICGs?) The TIMER database (https://cistrome.shinyapps.io/timer/) was employed to investigate the correlations between the 4 CRICGs and 6 types of ICI in the TME.

### 2.7. Screening for potential therapeutic agents and drug susceptibility testing (DST)

The Enrichr database (https://maayanlab.cloud/Enrichr/) was utilized to screen for SMDs that displayed therapeutic potential for NSCLC and had associations with the selected CRICGs. The Genomics of Drug Sensitivity in Cancer (GDSC) database (http://www.cancerrxgene.org/) was used in combination with the “ggplot2,” “ggpubr,” and “pRRophetic” packages to illuminate the differences between the high- and low-risk groups in susceptibility to targeted therapy and chemotherapy.

### 2.8. Statistical analysis

All statistical analyses were performed utilizing the R software (version 4.0.2). Intergroup comparisons were examined by the chi-square test or Fisher’s exact test. *P* < .05 indicated a difference of statistical significance.

## 3. Results

**Figure 1.**
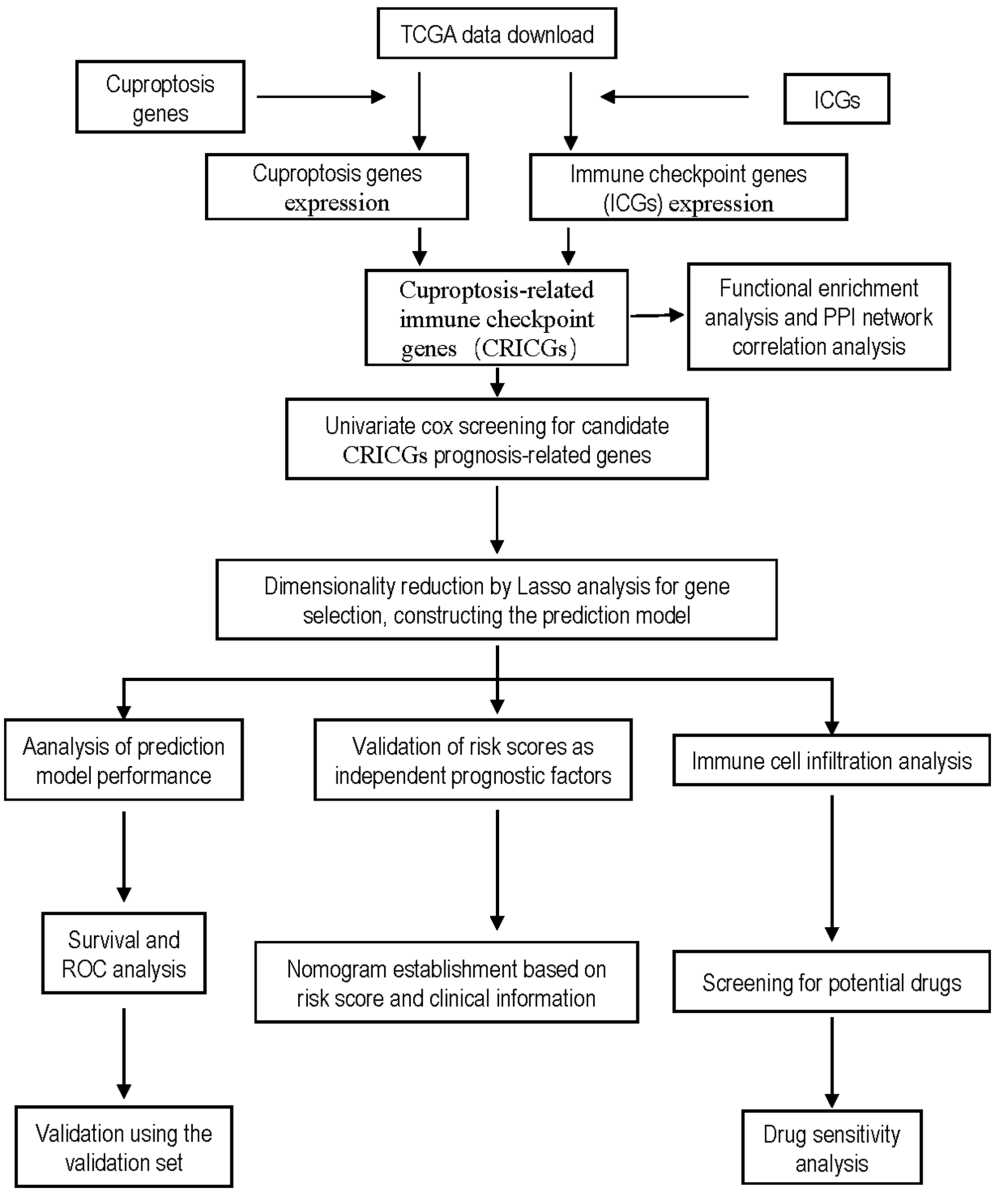
Flow chart of data collection and analysis. CRICG = cuproptosis-related immune checkpoint gene, ICG = immune checkpoint gene, ROC = receiver operating characteristic, TCGA = The Cancer Genome Atlas.

The study flow diagram is shown in Figure 1. The training dataset contained the mRNA expression and clinical data of 1041 patients with NSCLC (LUAD + LUSC) available in TCGA. The validation dataset was formed by the survival data of 233 NSCLC samples of the GSE30219 dataset from the GEO database.

### 3.1. GSEA and PPI network analysis of CRICGs

The NSCLC gene expression matrix that visualized the expression levels of 13 CRGs and 79 ICGs was downloaded from TCGA. Correlations between CRGs and ICGs were analyzed using the “limma” R package and 58 CRICGs were identified eventually.

### 3.2. GO and KEGG pathway enrichment analyses of potential functions of CRICGs

**Figure 2.**
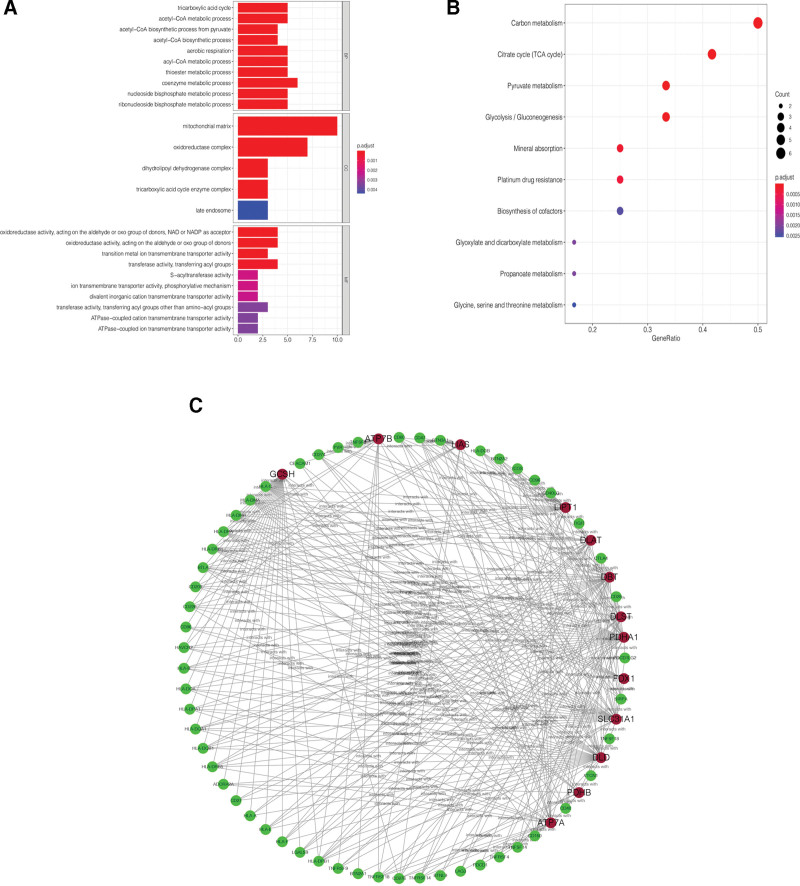
Enrichment analyses and protein–protein interaction network of differentially expressed CRICGs. (A) GO analysis; (B) KEGG analysis; and (C) protein–protein interaction network of differentially expressed CRICGs. CRICG = cuproptosis-related immune checkpoint gene, GO = Gene Ontology, KEGG = Kyoto Encyclopedia of Genes and Genomes.

Results of the GO enrichment analysis showed that under biological process terms, the 58 CRICGs were primarily enriched in the following processes: coenzyme metabolic process, tricarboxylic acid cycle, and acetyl-CoA metabolic process. Cellular component enrichment for all CRICGs was dominated by mitochondrial matrix, oxidoreductase complex, and dihydrolipoyl dehydrogenase complex. Enriched categories for molecular function included oxidoreductase activity, acting on the aldehyde or oxo group of donors, NAD or NADP as acceptor, oxidoreductase activity, acting on the aldehyde or oxo group of donors, and transition metal ion transmembrane transporter activity (see Fig. 2A). KEGG pathway enrichment for the 58 CRICGs was mainly found in the signaling pathways linked to the citrate cycle (tricarboxylic acid cycle), carbon metabolism, and pyruvate metabolism (see Fig. 2B).

The STRING database revealed that there were 58 nodes and 171 edges in the PPI network based on the 58 CRICGs. With an average local clustering coefficient of 0.611 and a PPI enrichment *P*-value < 1.0 e^−16^, the PPI network was constructed using Cytoscape (see Fig. 2C), where nodes in red and green colors represent CRGs and ICGs, respectively.

### 3.3. Identification of prognosis-related CRICGs and construction of predictive model

**Figure 3.**
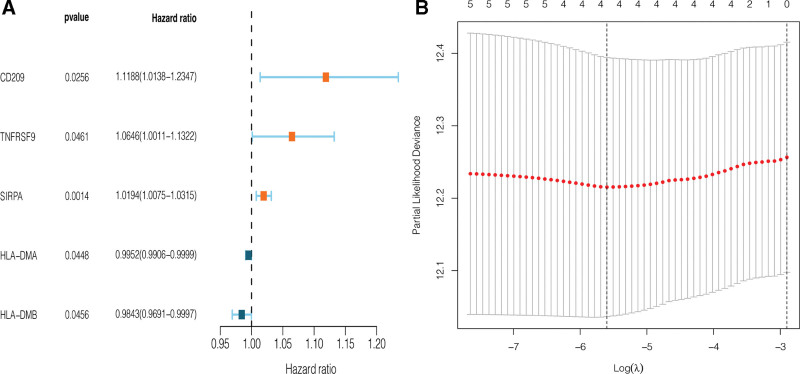
Identification of prognostic CRICGs by univariate Cox regression analysis and LASSO-penalized Cox regression analysis. (A) Forest plot shows candidate CRICGs. (B) LASSO regression analysis was used for dimensionality reduction for the 5 prognosis-associated CRICGs. *X* axis shows λ and *Y* axis shows error. CRICG = cuproptosis-related immune checkpoint gene, LASSO = least absolute shrinkage and selection operator.

A multivariate Cox regression model was utilized to assess potential associations of the 58 CRICGs with the overall survival (OS) of patients with NSCLC. Finally, 5 CRICGs were identified to have significant associations with NSCLC prognosis, including CD209, TNFRSF9, SIRPA, HLA-DMA, and HLA-DMB. On this basis, a forest plot was constructed as shown in Figure 3A. Following that, a predictive model of NSCLC prognosis was developed with least absolute shrinkage and selection operator Cox regression and 4 CRICGs, namely CD209, TNFRSF9, SIRPA, and HLA-DMB (see Fig. 3B). In the predictive model, the risk score is computed by the formula: risk score = (0.0827 × CD209 expression level) + (0.0778 × TNFRSF9 expression level) + (0.0167 × SIRPA expression level) + (−0.0296 × HLA-DMB expression level).

### 3.4. Performance evaluation of predictive model

**Figure 4.**
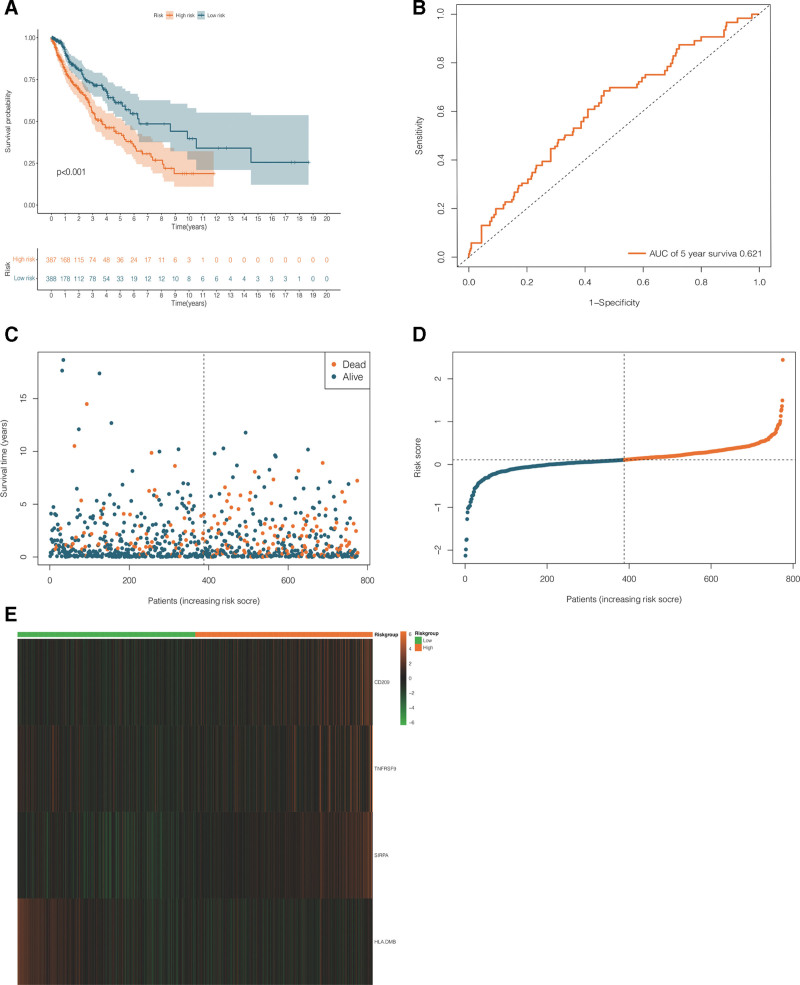
Performance evaluation of the predictive model based on cuproptosis-related immune checkpoint genes (CRICGs) using the training dataset. (A) The Kaplan–Meier (KM) survival analysis shows a significant difference in survival time between the high- and low-risk groups of non-small cell lung cancer (NSCLC) patients in the training dataset; (B) evaluation of predictive performance in the training dataset for 5-year overall survival (OS) based on time-independent receiver operating characteristic (ROC) analysis; (C) risk score system of NSCLC samples in the training dataset; (D) risk score distribution for NSCLC samples in the training dataset; and (E) heatmap of differences between the high- and low-risk groups in the expression levels of the 4 CRICGs in the training dataset. CRICG = cuproptosis-related immune checkpoint gene, NSCLC = non-small cell lung cancer, OS = overall survival, ROC = receiver operating characteristic.

NSCLC patients in the training dataset were classified into a high-risk group (n = 387) and a low-risk group (n = 388) using the median risk score as the cutoff point (see Fig. 4D). The KM survival curve indicated that survival time was significantly longer in the low-risk group than in the high-risk group (*P* < .001; see Fig. 4A). Moreover, the risk score system (Fig. 4C) also suggested a negative correlation between the risk score and NSCLC prognosis. The ROC curve (Fig. 4B) showed that the predictive model based on the 4 selected CRICGs demonstrated favorable efficiency and accuracy in predicting the 5-year survival of patients with NSCLC from TCGA (AUC_5_ = 0.621). Finally, a heatmap was generated to visualize the differences between the high- and low-risk groups in the expression levels of the 4 CRICGs (see Fig. 4E).

**Figure 5.**
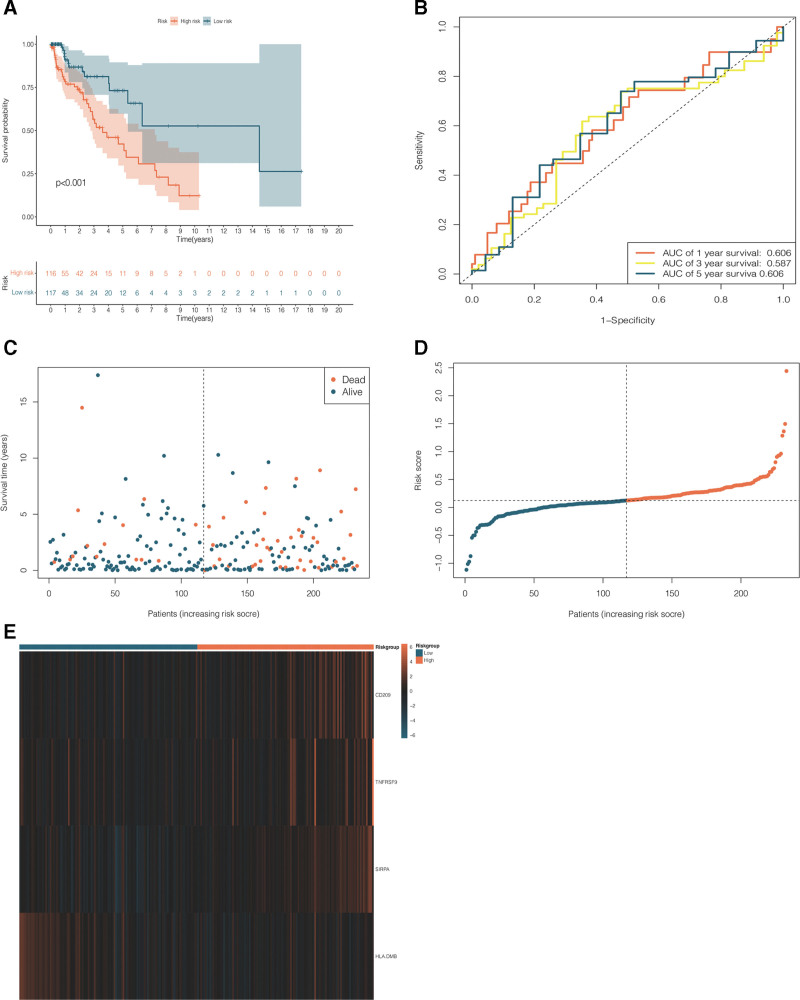
Performance evaluation of the CRICG-based predictive model using the validation dataset. (A) The KM survival analysis shows a significant difference in survival time between the high- and low-risk groups of NSCLC patients in the validation dataset; (B) evaluation of predictive performance in the validation dataset for 1-, 3-, and 5-year OS based on time-independent ROC analysis; (C) risk score system of NSCLC samples in the validation dataset; (D) risk score distribution for NSCLC samples in the validation dataset; and (E) heatmap of differences between the high- and low-risk groups in the expression levels of the 4 CRICGs in the validation dataset. CRICG = cuproptosis-related immune checkpoint gene, KM = Kaplan–Meier, NSCLC = non-small cell lung cancer, OS = overall survival, ROC = receiver operating characteristic.

The 4 CRICGs were assessed likewise for prognostic value using a GEO dataset for validation, with samples in the validation dataset being separated into a high-risk group (n = 116) and a low-risk group (n = 117) by the median risk score (see Fig. 5D). In line with the results yielded by analysis of the training dataset, the KM curve of the validation dataset suggested longer survival in the low-risk group (*P* < .001; see Fig. 5A). The risk score system displayed a negative correlation between the risk score and NSCLC prognosis (see Fig. 5C). As shown in the ROC curve, the predictive model delivered good performance, with AUC_1_ = 0.606, AUC_3_ = 0.587, and AUC_5_ = 0.606 (see Fig. 5B). Differences in the expression levels of the 4 CRICGs between the high- and low-risk groups were plotted as a heatmap (see Fig. 5E).

### 3.5. Risk score as an independent prognostic factor in NSCLC

**Figure 6.**
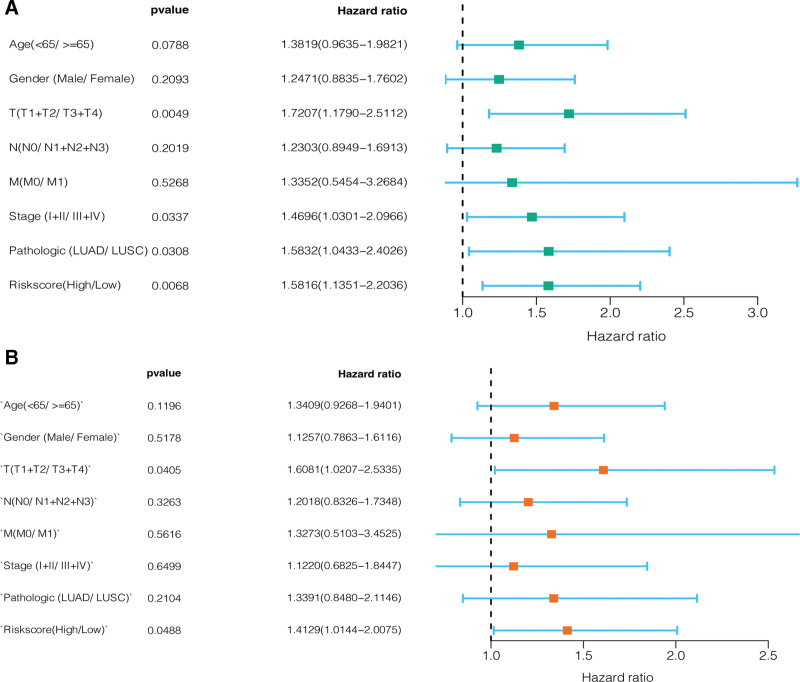
Validation of the risk score of the CRICG-based predictive model as an independent prognostic factor in NSCLC. (A) Univariate Cox regression analysis of the associations of the prognosis with the risk scores and clinical characteristics of NSCLC patients; (B) multivariate Cox regression analysis of the associations of the prognosis with the risk scores and clinical characteristics of NSCLC patients. CRICG = cuproptosis-related immune checkpoint gene, NSCLC = non-small cell lung cancer.

Uni- and multivariate Cox regression models were employed to assess the eligibility of the CRICGs that were used for constructing the predictive model as an independent prognostic factor in NSCLC. The univariate Cox regression analysis showed that the risk score, T staging, overall staging, and pathological patterns were strongly associated with the survival of patients with NSCLC (see Fig. 6A). The multivariate Cox regression analysis suggested that the risk score served as an independent prognostic factor for the survival of NSCLC patients (see Fig. 6B).

### 3.6. Associations between the predictive model and clinical characteristics of NSCLC patients

**Figure 7.**
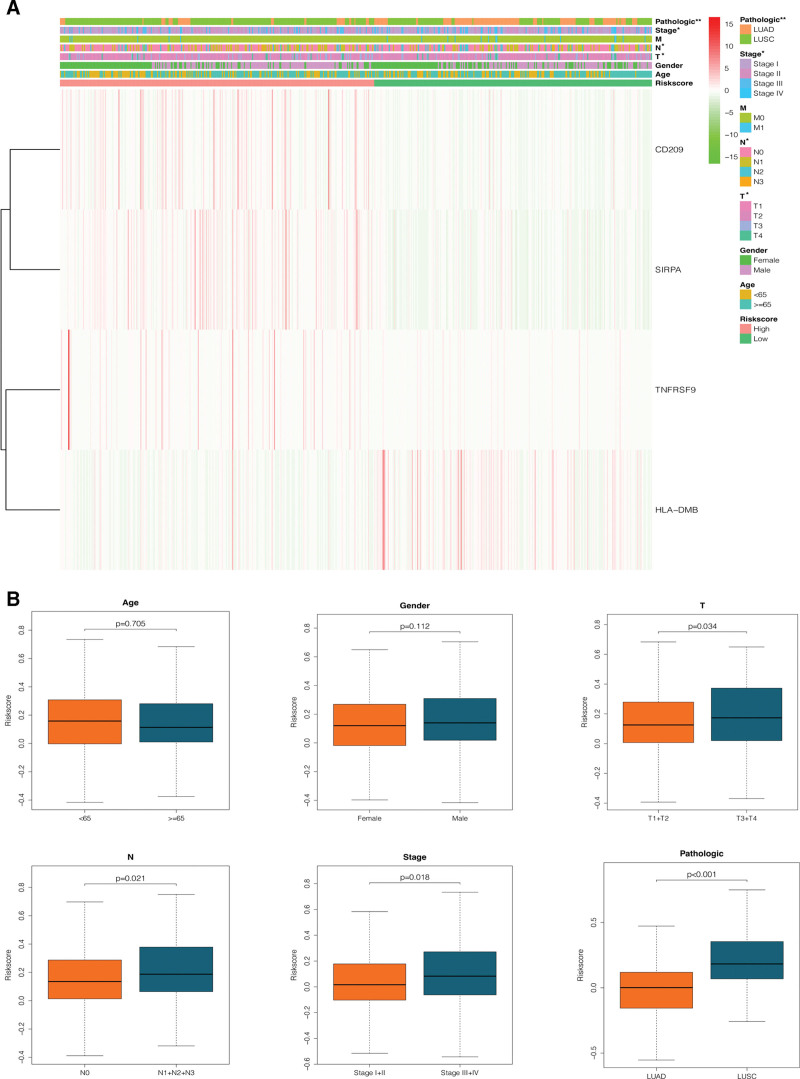
Correlation analysis between the risk scores and clinical characteristics of NSCLC patients. (A) Heatmap of correlations of clinical characteristics with high- and low-risk groups and the 4 CRICGs; (B) correlations between age, sex, T staging, N staging, overall staging, pathological patterns, and the risk score. CRICG = cuproptosis-related immune checkpoint gene, NSCLC = non-small cell lung cancer.

The chi-square and Fisher’s tests were conducted to investigate whether or not the 4 CRICGs applied to the predictive model were involved in NSCLC development and progression. Figure 7A, B demonstrates significant differences in T staging (*P* = .034), N staging (*P* = .021), and overall staging (*P* = .018) between the high- and low-risk groups, indicative of a higher risk score with the advancement of cancer stage at diagnosis; Moreover, there were significant differences in pathological patterns (*P* < .001), indicating a stronger likelihood of LUSC patients having a higher risk score than LUAD patients.

**Figure 8.**
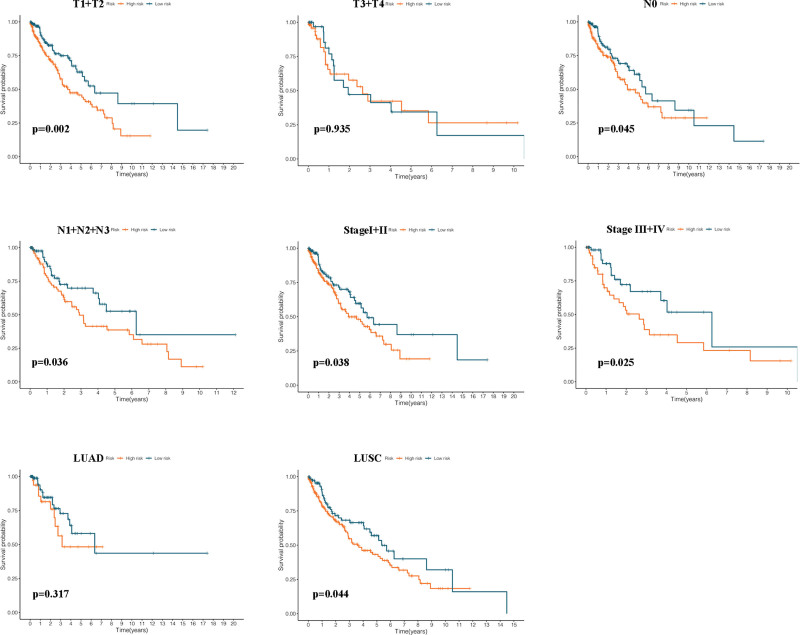
Kaplan–Meier curves of OS differences stratified by T stage, N stage, TNM stage and pathological classification between the high-risk groups and low-risk groups. TNM = tumor-node-metastasis.

Further, a subgroup analysis was carried out to investigate the associations between such CRICGs and NSCLC prognosis in each subgroup. The risk score of the predictive model delivered better performance in the T_1_ + T_2_ (*P* = .002), N_0_ (*P* = .045), N_1_ + N_2_ + N_3_ (*P* = .031), stage I + II (*P* = .038), stage III + IV (*P* = .025), and LUSC (*P* = .044) subgroups but was less predictive of prognosis in the LUAD and T_3_ + T_4_ subgroups (see Fig. 8).

### 3.7. Construction and performance evaluation of prognostic nomogram

**Figure 9.**
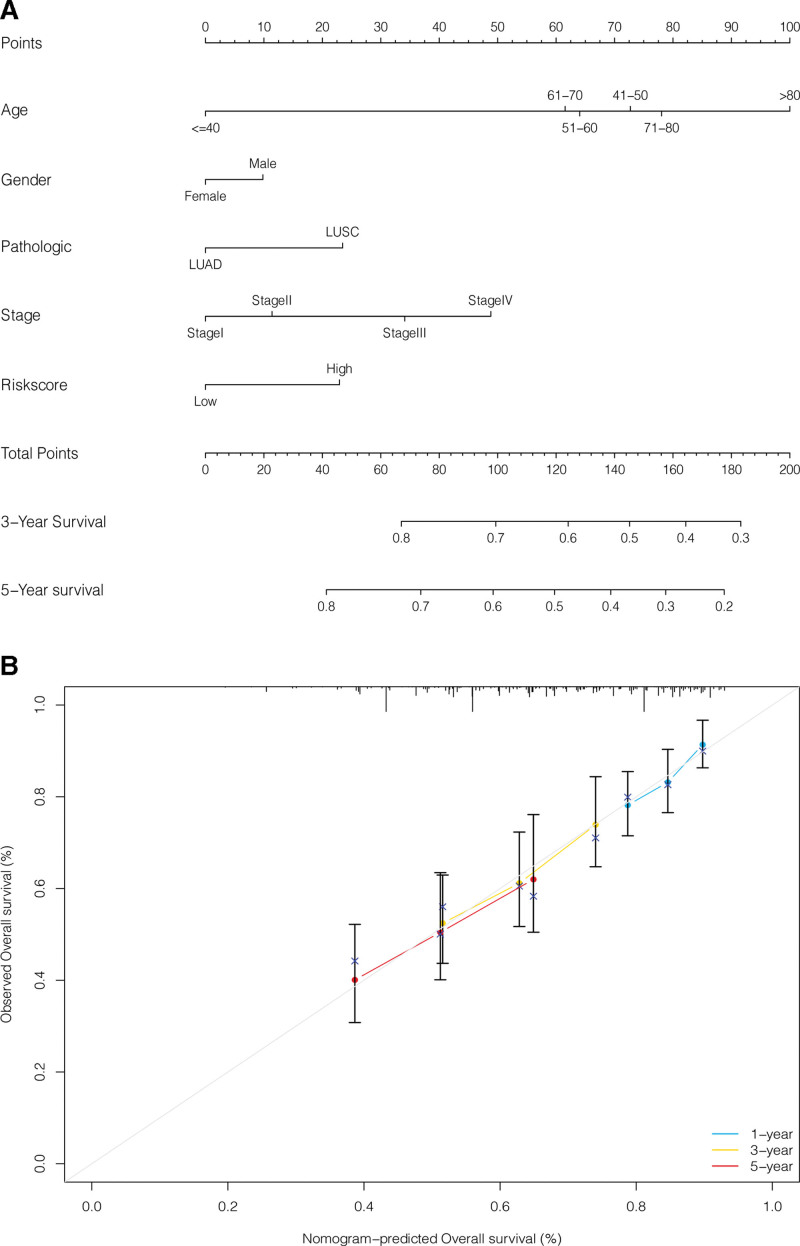
Construction and performance evaluation of nomogram. (A) Nomogram to predict 3- and 5-year survival in NSCLC patients. (B) Calibration curve to evaluate the predictive performance of the nomogram for 3- and 5-year survival in NSCLC patients. NSCLC = non-small cell lung cancer.

A prognostic nomogram was constructed using such parameters as age, sex, pathological patterns, overall staging, and the risk score of the predictive model to predict 3- and 5-year survival in individual NSCLC cases (see Fig. 9A). Further, the predictive performance of the nomogram was evaluated, and a calibration curve was created as shown in Figure 9B. The calibration curve represents the performance of an ideal prognostic nomogram, where 1-, 3-, and 5-year survival probabilities highly match with actual outcomes.

### 3.8. Gene set enrichment analyses

GSEA was performed to look further into potential differences between the high- and low-risk groups in mechanistic signaling pathways. The results revealed a predominance of tumor-related GnRH, Chemokine, ErbB, cAMP, and PPAR signaling pathways in the high-risk group; in the low-risk group, the GSEA showed enrichment for basal cell carcinoma, chemical carcinogenesis-DNA adducts, Hippo signaling pathway, Wnt signaling pathway, and breast cancer among all genes (see Supplementary Material 1, Supplemental Digital Content, https://links.lww.com/MD/R148).

### 3.9. Analysis of ICI in the TME

**Figure 10.**
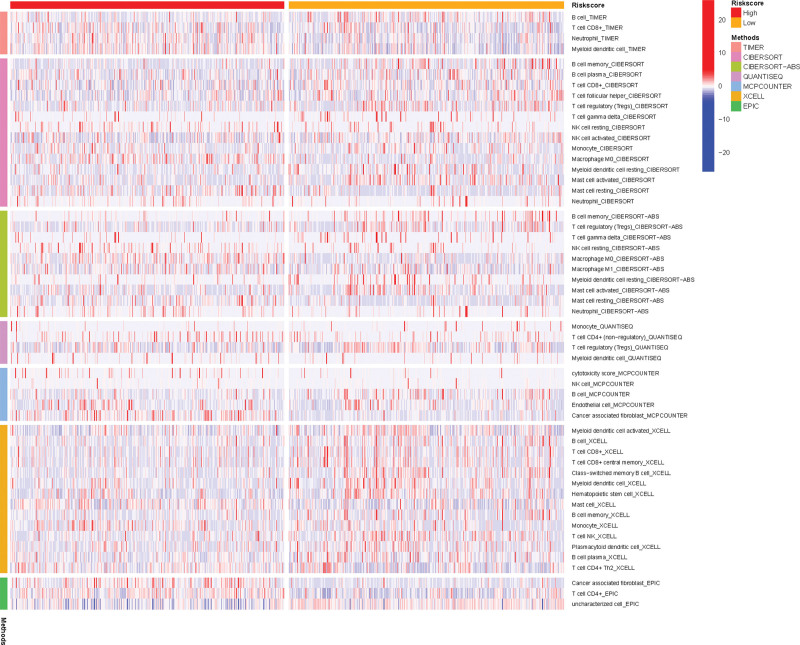
Different types of immune cell infiltration (ICI) in the tumor microenvironment (TME) in the high- and low-risk groups. ICI = immune cell infiltration, TME = tumor microenvironment.

Numerous types of ICI in the TME were discussed in this study to analyze their associations with the risk score of the predictive model. A heatmap of immune cells showing different infiltration levels in the high- and low-risk groups was generated by integrating 7 algorithms, including TIMER, xCell, CIBERSORT, CIBERSORT-ABS, MCP-counter, quanTIseq, and EPIC (see Fig. 10). The TIMER algorithm suggested that the low-risk group had a significantly higher infiltration level of B cells but substantially lower infiltration levels of CD8+ T cells, neutrophils, and myeloid dendritic cells than the high-risk group.

**Figure 11.**
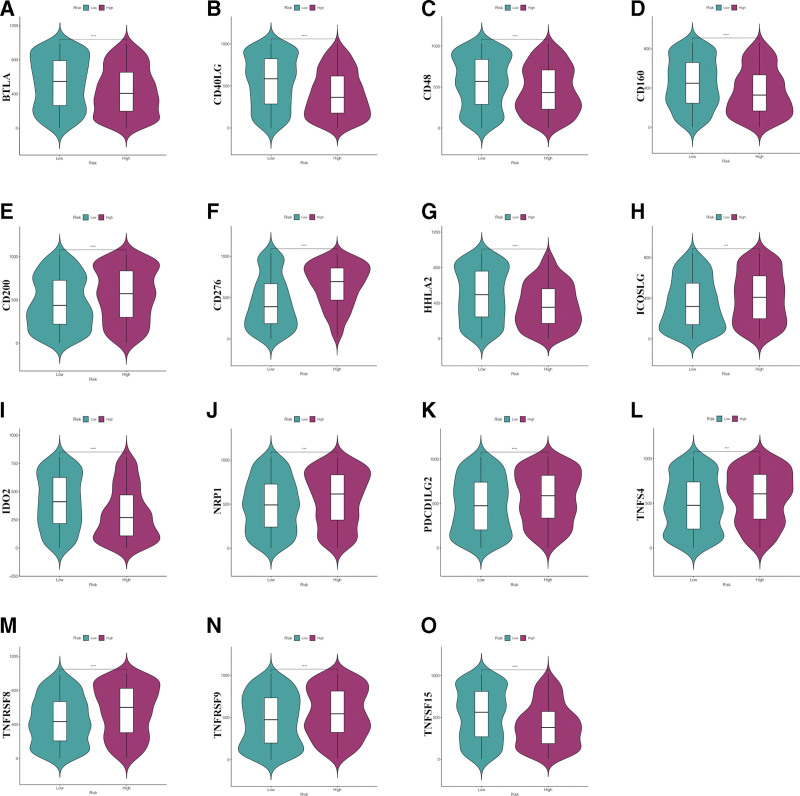
The relationship between risk score and immune checkpoints. (A–O) The relationship between BTLA, CD40LG, CD48, CD160, CD200, CD276, HHLA2, ICOSLG, IDO2, NRP1, PDCD1LG2, TNFS4, TNFRSF8, and TNFRSF9 TNFRSF15 expression levels and low-/high-risk group.

Given the importance of immune checkpoints in immunotherapy, this study also analyzed the associations between the risk score of the predictive model and the expression levels of 47 ICGs. As shown in Figure 11, BTLA, CD40LG, CD48, CD160, HHLA2, IDO2, and TNFRSF15 expression levels are significantly higher in the low-risk group; CD200, CD276, ICOSLG, NRP1, PDCD1LG2, TNFS4, TNFRSF8, and TNFRSF9 expression levels are significantly higher in the high-risk group.

**Figure 12.**
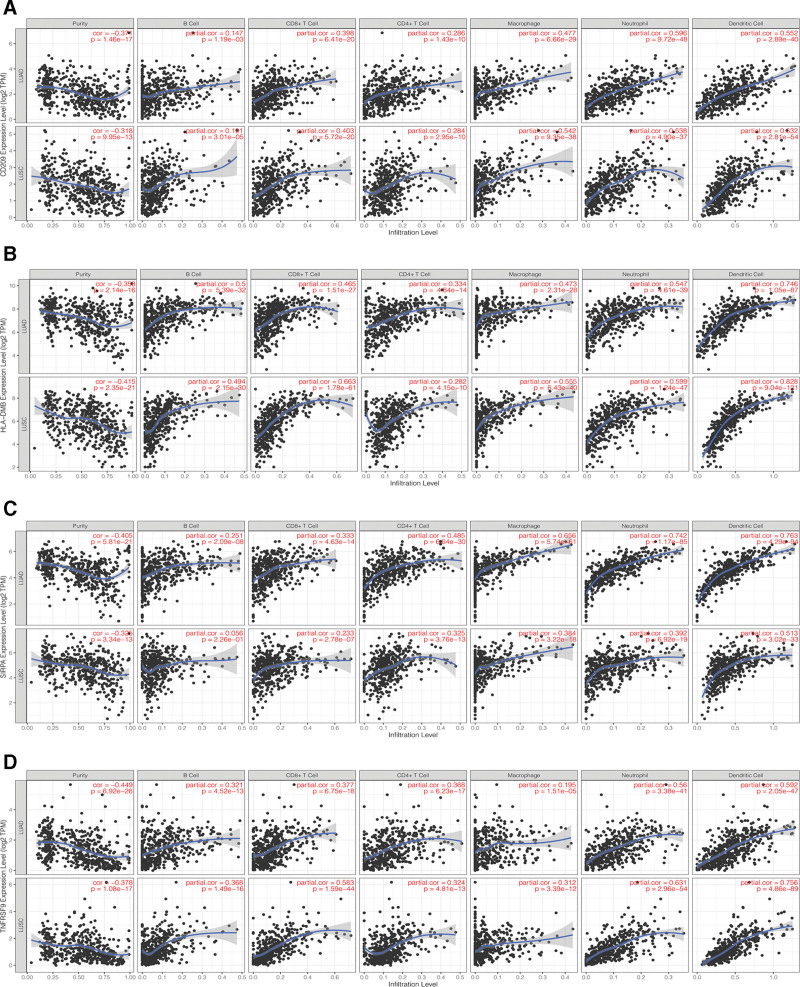
Analysis of associations between the 4 CRICGs and 6 types of ICI in the TME based on the tumor immune estimation resource (TIMER) database. (A–D) Associations of CD209, HLA-DMB, SIRPA, and TNFRSF9 expression with ICI levels of B cells, CD8+ T cells, CD4+ T cells, macrophages, neutrophils, and dendritic cells in the TME. CRICG = cuproptosis-related immune checkpoint gene, ICI = immune cell infiltration, TIMER = tumor immune estimation resource, TME = tumor microenvironment.

The TIMER database was used for the analysis of potential associations of the 4 CRICGs for constructing the predictive model with different types of tumor-infiltrating immune cells in the TME, including B cells, CD8+ T cells, CD4+ T cells, macrophages, neutrophils, and dendritic cells (see Fig. 12A–D).

### 3.10. Screening for potential therapeutic agents and DST

Eight SMDs were deemed to be the most promising candidates in the analysis of SMDs associated with the 4 CRICGs of the predictive model using the Enrichr database, including Fucose TTD 00008125, Epichlorohydrin CTD 00005906, Maltotetraose BOSS, Styrene oxide CTD 00000717, Alpha-d-Mannose TTD 00001913, Benzene CTD 00005481, Pemetrexed CTD 00003054, and 6-Deoxy-d-galactose BOSS (see Table 1).

**Table 1:** Eight small molecule drugs (SMDs) identified from the Enrichr database.

Term	Adjusted *P*-value	Combined score	Genes
Fucose TTD 00008125	.007	2204.785	CD209
Epichlorohydrin CTD 00005906	.007	2204.785	TNFRSF9
Maltotetraose BOSS	.04	1822.539	CD209
Styrene oxide CTD 00000717	.04	1822.539	TNFRSF9
Alpha-d-Mannose TTD 00001913	.043	1337.304	CD209
Benzene CTD 00005481	.043	164.892	HLA-DMB; SIRPA
Pemetrexed CTD 00003054	.047	1044.595	TNFRSF9
6-Deoxy-d-galactose BOSS	.047	1044.595	CD209

SMD = small molecule drug.

**Figure 13.**
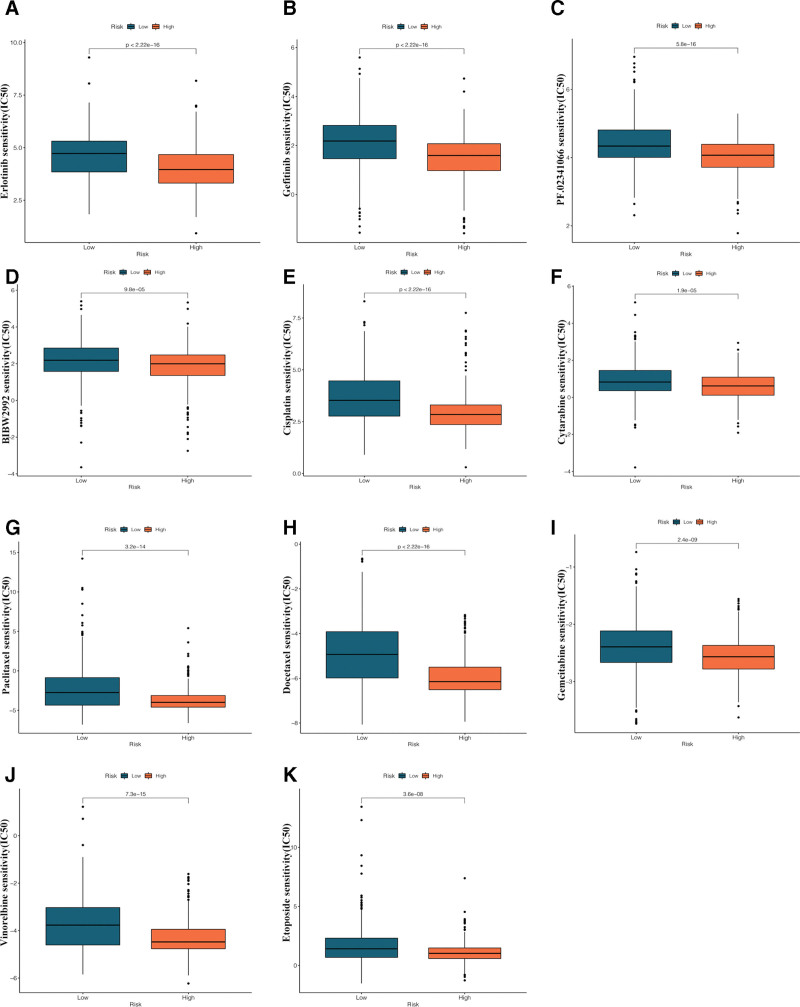
Drug susceptibility testing (DST) in the high- and low-risk groups. (A–K) Differences between the high- and low-risk groups in susceptibility of NSCLC to targeted drugs including erlotinib, gefitinib, PF.02341066 (crizotinib), BIBW2992 (afatinib), and common chemotherapy drugs including paclitaxel, docetaxel, gemcitabine, etoposide, and vinorelbine. DST = drug susceptibility testing, NSCLC = non-small cell lung cancer.

Drug susceptibility testing (DST) was conducted using the GDSC database to analyze potential differences between the 2 groups in susceptibility to 138 therapeutic agents. As shown in Figure 13, NSCLC is more susceptible to common targeted therapy and chemotherapy with erlotinib, gefitinib, PF.02341066 (crizotinib), BIBW2992 (afatinib), paclitaxel, docetaxel, gemcitabine, etoposide, and vinorelbine among the 11 most frequently used anticancer drugs. Moreover, the 50% inhibitory concentrations (IC50) were all higher in the high-risk group, suggesting higher resistance of NSCLC to these anticancer agents in the high-risk group.

## 4. Discussion

Lung cancer is the most common malignancy and the biggest cancer killer among men, and it is the second most prevalent and second leading cause of cancer deaths among women worldwide.^[1]^ Particularly, the vast majority of lung cancers are NSCLC.^[6,7]^ Given the recent breakthroughs achieved by the research on epidermal growth factor receptor-tyrosine kinase inhibitors and immune checkpoint inhibitors, NSCLC patients are provided with a wider range of treatment options and demonstrate significantly improved survival probabilities.^[8]^ However, drug resistance remains a real challenge leading to treatment failures. This requires further studies on interactions and mechanisms of substances in the TME to discover more effective therapeutic targets and prognostic biomarkers.

Cuproptosis, as a novel form of cell death, is gaining traction among researchers. Ample evidence shows that in malignant tumors, copper accumulation is associated with tumor cell proliferation and growth, metastasis, and angiogenesis in the TME.^[14,15]^ Immune checkpoint blockade has been heatedly discussed as a robust denominator approach to cancer therapy targeting immune checkpoint pathways. Cytotoxic T-lymphocyte-associated antigen 4, programmed death 1 (PD-1), and programmed death-ligand 1 are most extensively studied among all inhibitory receptors.^[16,17]^ Anti-cytotoxic T-lymphocyte-associated antigen 4 and PD-1/programmed death-ligand 1 antibodies like ipilimumab,^[18]^ pembrolizumab, and nivolumab^[19,20]^ have been approved by the US Food and Drug Administration for clinical application as immunotherapies.^[21]^ Despite the pronounced efficacy of such medications, there is still a large number of cancer patients who cannot benefit from immunotherapy. Therefore, it is essential to improve the diagnosis, treatment, and prognosis of lung cancer patients with more effective biomarkers.

This study focused on the role of CRICGs that combined cuproptosis and ICGs in predicting the prognosis and guiding the treatment of patients with NSCLC. The associations between 13 CRGs and 79 ICGs from open-source databases were analyzed to determine the expression levels of CRICGs. After univariate Cox and LASSO regression analyses, 4 prognosis-related CRICGs were identified and used for constructing the predictive model. Among these CRICGs, CD209 expression in peripheral blood mononuclear cells is shown to have a positive correlation with the treatment outcomes of patients with leukemic cutaneous T-cell lymphoma^[22]^; TNFRSF9 expression in regulatory T cells is found to be associated with NSCLC patients’ OS and response to anti-PD-1 immunotherapy, suggesting the immunomodulatory effect of TNFRSF9 on regulatory T cells in cancer patients^[23]^; SIRPA expression is proved to have a strong association with the prognosis and metastasis of colorectal cancer^[24]^; HLA-DMB in tumor cells is reported to play a role in the increase in tumor-infiltrating CD8+ T cells, and both are closely associated with the survival probabilities of patients with advanced serous ovarian cancer.^[25]^

In this study, NSCLC samples were stratified by the median risk score into high- and low-risk groups for performance evaluation of the predictive model in combination with KM curves, ROC curves, and a validation dataset. Then, the risk score was validated as an independent prognostic factor for OS of NSCLC patients. A prognostic nomogram was constructed using the risk scores and clinical data such as age, and TNM staging, which demonstrated ideal performance in predicting 3- and 5-year survival in NSCLC patients. According to the median risk score, NSCLC patients were assigned to high- and low-risk groups, and the GSEA results suggested enrichment for tumor-related pathways among the CRICGs. The ICI analysis further revealed the differences in infiltration levels of several types of immune cells between the high and low-risk groups. Eight CRICG-targeting SMDs with therapeutic potential were identified from the Enrichr database. Finally, the DST data from the GDSC database showed that the high-risk group appeared to demonstrate higher resistance to the frequently used NSCLC-targeted drugs and chemotherapy.

The present study has the following limitations: it has not investigated the underlying mechanism of CRICGs regulating the biological behavior of NSCLC cells; multicenter clinical cohorts are required to validate the utility of the predictive model.

## 5. Conclusions

In conclusion, the predictive model based on 4 selected CRICGs can accurately predict the prognosis of NSCLC patients. With the risk score of the predictive model being an independent prognostic factor for OS, NSCLC patients differing in immune responses and drug reactions can be divided into high- and low-risk groups by the risk score, offering new insights as to the clinical treatment and prognosis of NSCLC.

## Author contributions

**Data curation:** Ke Han.

**Formal analysis:** Ke Han.

**Methodology:** Ke Han.

**Software:** Ke Han.

**Supervision:** Ju Kun Wang, Jing Yao.

**Visualization:** Ju Kun Wang, Jing Yao.

**Writing – review & editing:** Jing Yao.

## Supplementary Material


