# Supplementary figures and images for: A study on the role of cuproptosis-related immune checkpoint genes in non-small cell lung cancer
Source: Medicine (Baltimore). 2026 Jan 16;105(3):e47163. doi: 10.1097/MD.0000000000047163 (PMC12826202; doi:10.1097/MD.0000000000047163)

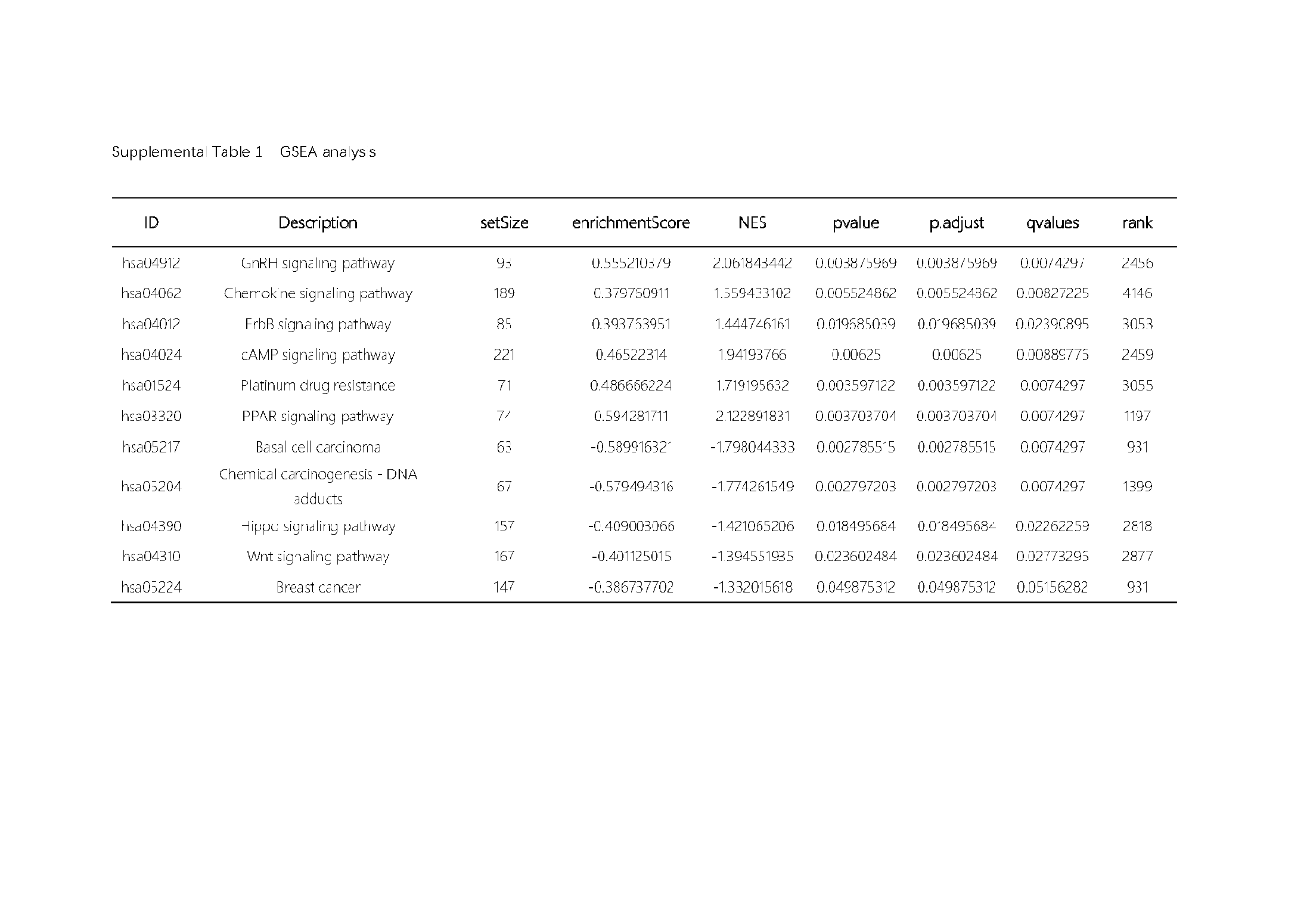

Supplement: Supplementary file 1 [file medi-105-e47163-s001.docx]
